# The Role of and Therapeutic Strategies for Eosinophils in Atopic Dermatitis

**DOI:** 10.3390/biomedicines14061212

**Published:** 2026-05-27

**Authors:** Guangyuan Cheng, Suting Sun, Guoshu Deng, Ying Luo, Miao Li, Hang Zhao, Xiaofan Yang, Ruiping Wang, Le Kuai, Ying Zhang, Bin Li, Yi Ru, Jiankun Song

**Affiliations:** 1 Shanghai Skin Disease Hospital, Institute of Dermatology, School of Medicine, Tongji University, Shanghai 200443, China; 2 Department of Dermatology, Yueyang Hospital of Integrated Traditional Chinese and Western Medicine, Institute of Dermatology, Shanghai University of Traditional Chinese Medicine, Shanghai 200437, China

**Keywords:** atopic dermatitis, eosinophils, inflammation, interleukin, chemoattractant cytokine ligand

## Abstract

Atopic dermatitis (AD) is a chronic inflammatory skin disease driven by immune dysregulation and epidermal barrier dysfunction, in which eosinophils act as key effector cells contributing to tissue damage and persistent inflammation. This comprehensive review elucidates the multifaceted contributions of eosinophils to the progression of AD. Driven by key type 2 cytokines (notably IL-4, IL-5, and IL-13) and specific chemokines, eosinophils infiltrate lesional skin and undergo IgE-mediated degranulation. The subsequent release of cytotoxic granule proteins, including major basic protein (MBP), eosinophil cationic protein (ECP), eosinophil-derived neurotoxin (EDN), and eosinophil peroxidase (EPX), directly induces keratinocyte apoptosis, exacerbates tissue remodeling, and sustains the local inflammatory cascade. Furthermore, we explore the intricate crosstalk between eosinophils and sensory neurons, which, alongside cytokines like IL-31, profoundly aggravates chronic pruritus. Consequently, modulating eosinophil activation and recruitment has emerged as a vital therapeutic approach. We systematically evaluate current and emerging pharmacological interventions, ranging from conventional topical corticosteroids to advanced targeted therapies. Particular emphasis is placed on the mechanistic impact of novel biologics and small-molecule Janus kinase (JAK) inhibitors, demonstrating how they attenuate eosinophilic inflammation. By identifying current gaps in this field, this review provides valuable insights for future research and clinical practice in the field of AD.

## 1. Atopic Dermatitis

AD is a prevalent chronic, relapsing inflammatory skin disease characterized by a compromised skin barrier and an overactive immune response leading to intense pruritus and increased sensitivity to external stimuli, accompanied by excessively dry, flaky skin and pronounced erythema [1]. Globally, AD has a high global prevalence, affecting approximately 15–20% of the pediatric population and up to 10% of adults [2]. Notably, about 50% of pediatric patients experience intermittent flare-ups or go on to develop other allergic disorders, such as asthma (AA) or allergic rhinitis (AR) during adulthood [3]. Furthermore, studies indicate that over 30% of individuals with atopic skin diseases experience significant psychiatric and psychosocial comorbidities [4]. In summary, patients with AD experience significant detrimental impacts on both their physical well-being and psychological health. However, the complex pathogenesis of AD hinders the development of targeted and effective treatments.

## 2. Eosinophils

The pathogenesis of AD is closely linked to immune dysregulation, particularly the abnormal activation of the Th2-type immune response [5]. Th2 cells in AD produce key cytokines such as IL-4 and IL-5, which are instrumental in increasing eosinophil levels and IgE production [6]. Elevated IgE levels and eosinophilia are hallmark features of AD and are directly associated with disease severity [7]. Eosinophils are granulocytes derived from hematopoietic stem cells in the bone marrow under the influence of IL-5, eotaxins, and other chemokines [8]. Characterized by their bilobed nuclei and intracellular granules, eosinophils are key effector cells in allergic inflammation and immune regulation. Their granules contain cytotoxic proteins, which can disrupt epithelial barriers, induce apoptosis, and drive localized tissue damage [9]. In AD, eosinophils play a role in regulating chronic inflammation and tissue damage.

Eosinophils amplify the Th2-type immune response by releasing cytokines such as IL-4, IL-5, and IL-13, contributing to skin barrier disruption and localized tissue injury and perpetuating immune dysregulation as the “atopic triad” [10]. AD, AR, and AA share a pathologic mechanism centered on a Th2-type immune response and eosinophil infiltration. AD often initiates the disease cascade, wherein skin barrier disruption facilitates allergen sensitization, triggering systemic type 2 inflammation that extends to the respiratory and gastrointestinal tracts, ultimately leading to allergic rhinitis and asthma [11,12]. Although recent studies have provided some insight, the relationship between eosinophils and AD remains to be thoroughly analyzed and summarized. Therefore, the main purpose of this study is to conduct a systematic review of the existing literature to elucidate the mechanisms by which eosinophils contribute to AD pathology and their clinical significance. It is hoped that this review will not only shed light on the role of eosinophils in AD but also inspire future researchers to delve deeper into this important area.

## 3. The Production, Recruitment, and Regulation of Eosinophils in AD

Extensive research has revealed that the distribution of eosinophils in both the peripheral blood and lesional skin of patients with AD is significantly elevated compared to that of healthy individuals [13,14]. Hence, as an important indicator of AD, the study of eosinophils’ production, recruitment, and regulation in AD is essential for guiding therapeutic strategies.

## 4. Eosinophil Production in AD

Eosinophils predominantly develop in the bone marrow, where they transform CD34^+^ eosinophil precursors (EoPs) under the influence of transcription factors. They eventually transform into mature eosinophils by the subsequent engagement of IL-5, IL-3, and granulocyte–macrophage colony-stimulating factor (GM-CSF) with their respective specific receptors [15,16]. In patients with AD, upregulation of the IL-5 receptor (IL-5R) in CD34^+^ hematopoietic progenitor cells within the bone marrow is observed. IL-5 exerts its action on eosinophils, thereby stimulating their production and activation [17].

## 5. Eosinophil Recruitment in AD

In AD patients and AD-like mouse models, IL-5 not only activates eosinophil production but also facilitates their chemotactic recruitment to sites of localized inflammation [18]. In response to various inflammatory factors and chemokines, eosinophils are mobilized from the bone marrow into the peripheral bloodstream [19]. Upon stimulation, eosinophils release a cascade of autocrine cytokines and chemokines [20], attracting more eosinophils to the inflamed site and perpetuating a vicious cycle. In addition, Ng, C.T. et al. [21] found that TNF-α can promote the chemotaxis and activation of inflammatory cells, increase vascular permeability, and indirectly promote the recruitment of eosinophils in AD. Thymic stromal lymphopoietin (TSLP), an epithelial cell-derived cytokine, exerts an important role in the recruitment of eosinophils in AD as well. One study injected TSLP subcutaneously into mice and observed diffuse infiltration of eosinophils [22]. This may be attributed to the upregulation of p-L-plastin by TSLP in AD, which, in conjunction with other cytokines, further augments the migration of eosinophils. In addition, human TSLP (hTSLP)-activated dendritic cells (DCs) prime CD4^+^ T helper cells to produce the proallergic cytokines IL-4, IL-5, IL-13, and TNF-α, while downregulating IL-10 and IFN-γ, which are central to eosinophil recruitment in AD [20].

In summary, IL-5 and other key cytokines drive eosinophil production, recruit eosinophils to inflamed sites, and perpetuate inflammation through autocrine signaling cascades and increased vascular permeability in the progress of AD. Additionally, TSLP promotes eosinophil infiltration via dual mechanisms: direct upregulation of p-L-plastin to enhance eosinophil migration and indirect induction of Th2 responses by modulating dendritic cell function, which further amplifies eosinophil recruitment.

## 6. The Regulation of Eosinophil Apoptosis in AD

It was discovered that serum, plasma, eosinophils, and eosinophil-stimulated supernatants from AD patients exhibit heightened concentrations of brain-derived neurotrophic factor (BDNF). Furthermore, in eosinophils derived from AD patients and stimulated by BDNF, the factor was observed to suppress eosinophil apoptosis [23]. Moreover, Ko et al. [24] have demonstrated that deficiency of the nuclear factor-κB kinase subunit β (IKKβ) in skin fibroblasts disrupts the canonical NF-κB signaling cascade, which in turn induces the overexpression of chemoattractant cytokine ligand (CCL)11, thereby precipitating an escalation in eosinophil infiltration within the early phases of dermal inflammation in AD-like mice models. Wedi et al. [25] also found that IL-3, IL-4, IL-5, and GM-CSF can inhibit the demise of eosinophils, whereas corticosteroids can induce apoptosis in eosinophils. In addition, Schwartz et al. [26] found that IL-5 upregulates the anti-apoptotic protein Bcl-x_L_, which in turn prevents eosinophil apoptosis. In addition, Beyer, L. et al. [27] discovered that eosinophils from AD patients express the histamine H4 receptor (H_4_R) robustly, from which the histamine can activate human eosinophils. Concurrently, the histamine’s interaction with both H_2_R and H_4_R leads to the upregulation of IL-18/IL-18Rα expression, culminating in the activation of eosinophils [27]. In short, various factors such as BDNF, cytokines (IL-3, IL-4, IL-5, GM-CSF), and histamines can inhibit eosinophil apoptosis, while corticosteroids induce eosinophil apoptosis, thereby influencing the inflammatory processes in atopic dermatitis.

## 7. Eosinophil Products in AD

**Figure 1:**
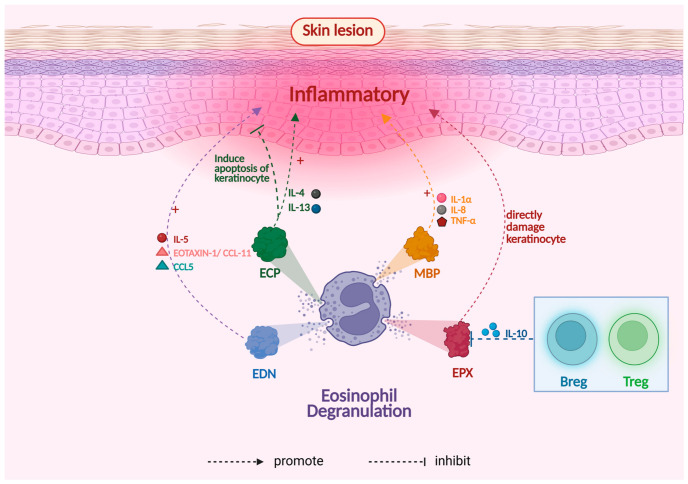
The role of MBP, ECP, EDN, and EPX in AD inflammation. This schematic illustrates the pathogenic mechanisms of the four major eosinophil granule proteins (MBP, ECP, EDN, EPX) in AD. Upon degranulation in the dermis, MBP directly damages keratinocyte membranes and activates keratinocytes to secrete pro-inflammatory cytokines (IL-1α, IL-8, TNF-α), amplifying the local inflammatory cascade. ECP induces keratinocyte apoptosis, disrupts the epidermal barrier, and stimulates Th2 cytokine release. EDN induces cutaneous inflammation by upregulating IL-5, eotaxin-1, and CCL5 in keratinocytes. EPX directly damages keratinocyte and its secretion is negatively regulated by IL-10 from regulatory B cells (Bregs) and T cells (Tregs). Collectively, these granule proteins drive epidermal barrier disruption, persistent inflammation, tissue remodeling, and pruritus in AD. MBP: major basic protein; ECP: eosinophilic cationic protein; EDN: eosinophilic-derived neurotoxin; EPX: eosinophilic peroxidase; Breg: regulatory B cells; Treg: regulatory T cells; IL: interleukin; CCL: chemoattractant cytokine ligand. Created in BioRender. Luo, Y. (2026) https://BioRender.com/9ourhe3 (accessed 30 April 2026).

Eosinophils are involved in the pathological process of AD by releasing a variety of granule proteins with cytotoxic and pro-inflammatory properties (Figure 1), such as major basic protein (MBP), eosinophilic cationic protein (ECP), eosinophilic-derived neurotoxin (EDN), and eosinophilic peroxidase (EPX) [28].

### 7.1. MBP

MBP is one of the most important proteins in eosinophil granules and has been widely studied for its cytotoxic and pro-inflammatory properties [29]. Immunofluorescence analysis of skin tissue from patients with AD revealed the presence of MBP, despite the absence of eosinophils in biopsy samples [30]. Accordingly, eosinophils undergo MBP degranulation within the dermis of patients with AD. MBP exerts its pathogenic effects through direct damage to epithelial cells and other tissue structures. Studies have shown that MBP activates keratinocytes, prompting them to secrete cytokines such as IL-1α, IL-8, and TNF-α. These cytokines not only amplify the local inflammatory response but also orchestrate a cascade of events that recruit and activate various immune cells, including neutrophils, lymphocytes, and macrophages, further fueling inflammatory events and exacerbating pathological changes in AD [31]. This indicates that the interplay between MBP and keratinocytes, along with the ensuing cytokine production, exerts an important influence on the inflammatory processes in AD.

Collectively, MBP mediates AD pathogenesis through direct tissue damage and keratinocyte-dependent cytokine release, leading to persistent immune activation and chronic inflammation.

### 7.2. ECP

ECP is a key protein released by eosinophils, with dual roles in antimicrobial defense and tissue damage, particularly in allergic diseases [32]. In AD, elevated ECP levels contribute to disease pathology by inducing keratinocyte apoptosis, which compromises the skin barrier, facilitating allergen and pathogen entry [33]. ECP also stimulates the release of cytokines such as IL-4 and IL-13, amplifying allergic inflammation, disrupting extracellular matrix function, and increasing vascular permeability and edema [33,34]. ECP exacerbates AD progression through keratinocyte apoptosis-induced barrier disruption and cytokine-driven amplification of allergic inflammation, extracellular matrix dysfunction, and vascular leakage. Studies have demonstrated a close correlation between elevated ECP levels and clinical symptoms of AD, such as itching and erythema. ECP further intensifies disease severity by promoting eosinophil aggregation and activation at lesion sites, enhancing local immune responses and prolonging chronic inflammation [35]. These processes not only impair the structural integrity of the skin but also perpetuate the inflammation.

To sum up, ECP mediates epidermal barrier collapse via keratinocyte apoptosis while propelling Th2-skewed immunity and eosinophil-driven inflammation, culminating in multifaceted tissue remodeling and clinical exacerbation.

### 7.3. EDN

EDN is a protein with neurotoxic and pro-inflammatory properties [36]. Studies have shown that serum EDN levels are significantly higher in patients with severe refractory AD and severe AD compared to those with mild-to-moderate AD and control groups. Higher EDN levels may correlate with increased disease severity in AD. In addition, patients with higher EDN levels are more likely to experience disease recurrence [37], suggesting that it can be used as a potential biomarker for predicting the recurrence of AD. Gomułka et al. [38] found that the levels of EDN and vascular endothelial growth factor (VEGF) in patients with AD were significantly higher than those in the healthy control group, and that EDN level was positively correlated with SCORAD score. Moreover, it was discovered that EDN induces cutaneous inflammation by upregulating IL-5, eotaxin-1, and CCL5 in keratinocytes (mRNA/protein levels) via MMP9-independent pathways, concurrently triggering mitochondrial apoptosis and ROS production [39]. These findings suggest its potential as a therapeutic target in eosinophil-rich dermatoses like AD. Thus, EDN may exacerbate AD symptoms by promoting angiogenesis and enhancing skin inflammatory response.

Consequently, elevated EDN demonstrates a predictive association with AD severity and relapse, with pathological interactions involving VEGF-driven vascular remodeling and inflammatory cascade potentiation.

### 7.4. EPX

EPX is a heme-containing peroxidase enzyme [40] that is primarily synthesized and stored in eosinophil granules and is released upon cellular activation [41]. Compared with the healthy control group, the level of EPX was significantly elevated in the AD patient group [42]. Moreover, the presence of IgE autoantibodies targeting EPX in patients with severe AD reveals its immunomodulatory role [43], indicating its potential as a future therapeutic target. EPX can induce inflammatory responses and damage normal tissues. It can also stimulate the degranulation of mast cells and the release of histamine in mice, playing a crucial role in the inflammatory process of AD. In addition, studies have shown that IL-10^−/−^ Breg cells promote the activation of eosinophils and their infiltration into skin tissues, thereby aggravating the symptoms of AD [44]. Evidently, regulatory B cells (Breg cells) inhibit the secretion of EPX by eosinophils in an IL-10-dependent manner.

Collectively, EPX is a marker of eosinophil activation in AD, linked to inflammation and disease progression, with potential as a therapeutic target.

## 8. The Interaction of Eosinophils and Molecules in AD

### 8.1. Eosinophils and Cytokines

Within the context of AD, IL-4 and IL-13 are prominent in the Th2-type inflammatory response, leading to a reduction in skin barrier protein expression and concurrently promoting eosinophil infiltration into inflamed regions [3]. Meanwhile, IL-4 and IL-13 upregulate VCAM-1 expression on endothelial cells, thereby facilitating eosinophil tethering and extravasation into inflamed skin and promoting collagen deposition [45]. It has been found that eosinophils promote the expression of IL-31 [46]. Cheung et al. [47] cultured eosinophils and keratinocytes individually and found that a co-culture system with the addition of IL-31 significantly promoted the release of the pro-inflammatory factors IL-1β and IL-6 and the chemokines CXCL1, CXCL8, CCL2, and CCL8 from eosinophils. Consequently, it was concluded that IL-31 activates the interaction between eosinophils and keratinocytes, leading to the release of cytokines and chemokines. In addition to IL-31-mediated pathways, direct crosstalk between eosinophils and cutaneous sensory neurons plays a key role in the pathogenesis of chronic pruritus in AD. Eosinophils function as potent neuromodulators by synthesizing and releasing specific neurotrophins, particularly nerve growth factor (NGF) and BDNF. NGF binds to the high-affinity TrkA receptor on sensory nerve fibers, promoting marked nerve hyperinnervation in the epidermis and significantly lowering the itch threshold [48]. Furthermore, eosinophil-derived granule proteins can directly activate sensory neurons. Proteases and cationic proteins such as MBP and ECP have been shown to cleave and activate protease-activated receptor 2 (PAR2) on peripheral nerve endings. This PAR2 activation does not depend on traditional histamine pathways but instead transmits potent signals directly to the central nervous system, thereby exacerbating chronic pruritus in AD [49].

Group 2 innate lymphoid cells (ILC2s) are a subset of lymphocytes which can produce type 2 cytokines [50]. IL-25, IL-33, and TSLP are cytokines that activate ILC2 and are highly expressed in AD [51]. In AD-like mice models, ILC2 secretes IL-5, IL-13, and CCL11 to amplify their systemic and cutaneous expression. Meanwhile, eosinophils and ILC2 interact to jointly produce fibrosis-inducing cytokines (IL-13, CTGF, TGF-β1), intensifying AD inflammation and driving epidermal thickening [52]. It has been demonstrated that eosinophils and Th2 cells both secrete IL-5, and epithelial cell-derived IL-25 further promotes IL-25 receptor-expressing Th2 cells to enhance IL-5 secretion; this cytokine network is reinforced by ILC2-derived IL-5, which synergizes with eosinophil-derived factors to amplify local secondary allergic immune responses [53].

TGF-β1 and IL-11 are key factors that mediate tissue repair and remodeling. During the acute phase of AD, the expression of IL-17 is markedly augmented [14,54]. The upregulation of IL-17 in skin lesions stimulated eosinophils to produce pro-fibrotic cytokines, such as TGF-β11 and IL-11, which promote COL1 and COL3 deposition and stimulate fibroblasts to synthesize and secrete collagen, resulting in thickening of the epidermis [55].

### 8.2. Eosinophils and Chemokines

Eotaxin-1/CCL11, eotaxin-2/CCL24, and eotaxin-3/CCL26 are CC chemokines that are functional ligands for CC chemokine receptor 3 (CCR3), which is preferentially expressed in eosinophils [56]. CCL5 is a chemokine for eosinophils and lymphocytes. It can also be produced by eosinophils via autocrine signaling, mediating the infiltration of eosinophils into the fibroblast layer of the dermis and exacerbating the inflammatory response in AD. CCL17 and CCL22 are members of the CC family of Th2-type chemokines [57]. In the peripheral blood of AD patients, the expression of CCL17 and CCL22 was proportional to the number of eosinophils in the serum, which may be due to the fact that CCL17 and CCL22 exacerbate the inflammatory response by recruiting Th2 cells to the site of inflammation through their interaction with the chemokine receptor CCR4. Mc Aleer et al. [58] detected high CCL17 expression in the plasma and skin stratum corneum of infantile AD patients and demonstrated that CCL17 expression levels were significantly correlated with SCORAD scores, suggesting that CCL17 may serve as a potential marker for AD severity assessment. Islam et al. [59] demonstrated that CCL8, a CC chemokine, significantly amplifies eosinophilic inflammation in wild mice. CCL8^−/−^ mice showed reduced inflammation, highlighting its role in Th2-type skin inflammation through the CCL8-CCR8 pathway. This pathway triggers IL-5 and IL-25 release, promoting eosinophil migration and enhancing allergic immune responses via reciprocal regulation between eosinophils and Th2 cells.

To summarize, CCL5, CCL8, CCL11, CCL17, CCL22, CCL24, and CCL26 primarily augment the inflammatory response in AD by either directly or indirectly inducing the recruitment or expression of eosinophils.

### 8.3. Eosinophils and Adhesion Molecules

Aberrant expression of intercellular adhesion molecules in the skin of AD patients often contributes to the initiation and escalation of inflammation. These adhesion molecules are important for the migration and activation of immune cells. Bruijnzeel et al. [60] identified that the expression of E-selectin, VCAM-1, and ICAM-1 is upregulated in AD skin lesions, exacerbating the inflammatory response by recruiting eosinophils to the affected areas. Consequently, the dysregulated expression of these molecules may precipitate the mislocalization and activation of immune cells, thereby inducing skin inflammation in AD. In addition, studies have revealed that eosinophils in the peripheral blood of AD patients exhibit high levels of the FH6 antigen and a pronounced affinity for soluble P-selectin [61]. P-selectin, in turn, binds to P-selectin glycoprotein ligand 1 (PSGL-1), a ligand on eosinophils, facilitating selective leukocyte migration in AD patients [62]. Except for the above adhesion molecules, increased expression of platelet factor 4 (PF4) also induces eosinophils to migrate to skin lesions in AD patients [63].

### 8.4. Eosinophils and IgE

In AD patients, IgE-mediated eosinophil activation is indirectly facilitated through mast cells, as mast cell degranulation precedes eosinophil infiltration into new lesions [64]. When cell-bound IgE binds to allergens, it sensitizes mast cells in the skin, ultimately leading to the production of inflammatory mediators, cytokines (including IL-4, IL-5, IL-13, and TNF-α), and chemokines, which promote eosinophil infiltration [65]. Moreover, eosinophils in AD lesions express FcεRI (high-affinity IgE receptor) and CD23 (FcεRII), enabling direct binding of IgE–allergen complexes [65]. This interaction triggers eosinophil degranulation via Syk/PLCγ2 signaling, releasing cytotoxic proteins (e.g., EPX, MBP) [66]. Clinically, the IgE inhibitor omalizumab is also commonly used to treat allergic diseases. Recently, a clinical trial proved that the serum levels of both IgE and eosinophils were decreased after treatment with omalizumab [67].

**Figure 2:**
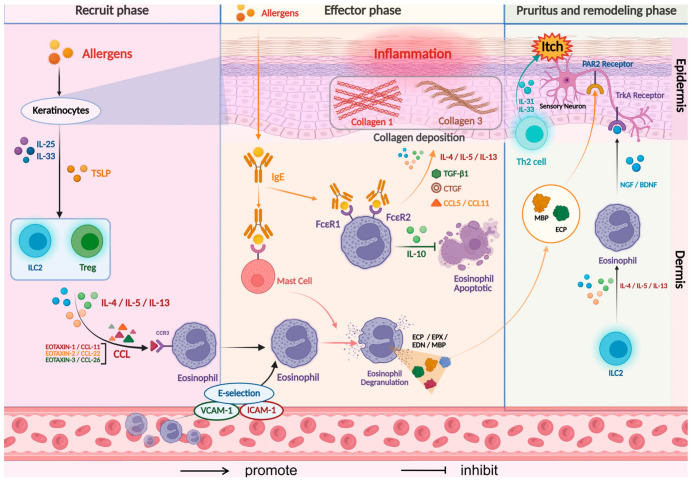
The interaction of eosinophils and molecules in AD. (1) Recruitment phase: Allergens penetrating the impaired epidermal barrier trigger keratinocyte release of TSLP, IL-33, and IL-25, which activate ILC2 and Th2 cells to secrete IL-5 and chemoattractants (eotaxins/CCL11, CCL5, CCL26). These chemokines bind CCR3 on eosinophils, directing their migration from circulation into the dermis via adhesion molecules (VCAM-1, ICAM-1, E-selectin). (2) Effector phase: IgE–allergen complexes crosslink FcεRI on mast cells, inducing degranulation and further eosinophil chemotaxis. Recruited eosinophils release cytotoxic granule proteins (MBP, ECP, EDN, EPX) causing keratinocyte apoptosis and barrier disruption, while secreting IL-4, IL-13, TGF-β1, and CTGF that amplify Th2 inflammation and drive fibrosis/collagen deposition. (3) Pruritus and remodeling phase: Eosinophil-derived NGF activates TrkA on sensory neurons inducing hyperinnervation, while MBP and proteases activate PAR2 to signal itch independently of histamine. IL-31 from Th2 cells synergizes with IL-33 to activate eosinophil–fibroblast crosstalk, perpetuating chronic pruritus and tissue remodeling. TSLP: Thymic stromal lymphopoietin; Breg: regulatory B cells; Treg: regulatory T cells; CCL: chemoattractant cytokine ligand; CCR3: CC chemokine receptor 3; CTGF: connective tissue growth factor; FcεRI: high-affinity IgE receptor; FcεRII/CD23: low-affinity IgE receptor; ICAM-1: intercellular adhesion molecule 1; VCAM-1: vascular cell adhesion molecule 1; MBP: major basic protein; ECP: eosinophilic cationic protein; EDN: eosinophilic-derived neurotoxin; EPX: eosinophilic peroxidase; IgE: immunoglobulin E; IL: interleukin; ILC2: group 2 innate lymphoid cells; PF4: platelet factor 4; TGF-β1: transforming growth factor-beta 1; Th2: T helper 2 cell; TRPV1: transient receptor potential vanilloid 1;. BDNF: brain-derived neurotrophic factor; NGF: nerve growth factor; PAR2: protease-activated receptor 2; Created in BioRender. Luo, Y. (2026) https://BioRender.com/o07o5b8 (accessed 30 April 2026).

To sum up, during the initial phase of AD, extrinsic antigens bind to IgE receptors on mast cells, triggering the release of eosinophil chemotactic factors, which leads to an increase in eosinophils (Figure 2).

## 9. Therapeutic Approaches Affecting Eosinophils in AD: Direct and Indirect Mechanisms

The role of eosinophils in AD has been increasingly recognized and various therapeutic strategies have been explored to modulate their activity or reduce their impact on disease progression. While many treatments affect multiple cell types and pathways, their influence on eosinophils can be indirectly observed through changes in blood or tissue eosinophil counts. This section discusses therapeutic approaches that have been associated with alterations in eosinophil biology.

### 9.1. Glucocorticosteroids

Topical glucocorticoids (TCSs) remain the first-line therapy for moderate-to-severe AD due to their multifaceted anti-inflammatory effects [68,69]. Glucocorticoids inhibit eosinophil function and the production of upstream cytokines that stimulate eosinophils [70,71,72] (including IL-5, CCL5, etc.), blocking the pathways of eosinophil recruitment and activation [73]. They exert their effects by transcriptionally repressing key eosinophil-associated cytokines, including IL-5 and GM-CSF [74], while simultaneously reducing cutaneous expression of eosinophil chemotactic factors such as CCL5 and CCL11 [73].

In addition, they disrupt eosinophil recruitment networks by blocking α4β1 integrin-mediated adhesion to VCAM-1 and downregulating CCR3 chemokine receptor expression [75]. Commonly used topical hormones include hydrocortisone cream, furoate mometasone cream, clobetasol propionate cream, etc. [76]. In addition to anti-inflammatory effects, these drugs can alleviate the symptoms of AD to some extent by inhibiting eosinophil expression or reducing their immune response.

### 9.2. Antihistamines

Eosinophils from AD patients highly express histamine H4 receptor (H_4_R), while histamine H1 receptor (H_1_R) is predominantly expressed on immune cells (e.g., macrophages, dendritic cells, and keratinocytes) and endothelial cells in AD lesions. Histamine can activate human eosinophils via H_4_R. Meanwhile, histamine also binds to histamine 2 receptor (H_2_R) and histamine 4 receptor (H_4_R), which in turn upregulates IL-18/IL18-Rα expression and ultimately promotes eosinophil activation. Ling et al. [77] demonstrated that H_4_R induced the upregulation of adhesion molecules CD11b/CD18 (Mac-1) and CD54 (ICAM-1) on eosinophils. In addition, antihistamines can inhibit the migration/degranulation of eosinophils. Notably, antihistamines exert dual effects on eosinophils’ biology: H_4_R antagonists directly inhibit eosinophil migration and degranulation [78], while H_1_R antagonists target H_1_R-expressing non-eosinophil cells (e.g., keratinocytes and macrophages) to suppress the secretion of GM-CSF, TNF-α, and IL-8 [79]. These cytokines are critical for eosinophil recruitment from the bone marrow and local activation in AD lesions. Common FDA-approved H_1_R antagonists used to manage AD-related pruritus include cetirizine and loratadine [80,81], while H_4_R antagonists like izuforant remain in investigational stages [82]. Collectively, administration of antihistamines (targeting H_1_R and/or H_4_R) effectively diminishes eosinophil production, aggregation, and pro-inflammatory activity, thereby alleviating AD-related inflammation.

### 9.3. Immunosuppressants

Cyclosporin A, azathioprine, and methotrexate are conventional systemic immunosuppressive agents recommended by clinical guidelines for the management of moderate-to-severe refractory AD [83]. Among them, AD patients have overexpression of CD11b in eosinophils, and treatment of AD patients with cyclosporin A results in improvement of clinical symptoms as well as a reduction in CD11b expression [84,85]. During the treatment of AD patients, we usually use calcineurin inhibitors (e.g., Tacrolimus) [70], which share a similar mechanism of action with glucocorticoids. In AD patients, tacrolimus application significantly reduces the number of skin cells that are immunoreactive to eosinophil-attracting ligands (IL-5, IL-8, CCL5) and the eosinophil-expressed receptor CCR3, which mediates eosinophil recruitment by binding to its ligands (e.g., CCL5, CCL11) in AD lesions [86]. Moreover, Interferon-alpha (IFN-α) is another therapeutic option for AD. Teijaro, J. R. et al. [87] found that IFN-α signaling enhances IL-10 secretion (a key anti-inflammatory cytokine that suppresses eosinophil activation and survival) while directly inhibiting IL-5 transcription. These dual effects augmented IL-10-mediated suppression and reduced IL-5-driven stimulation, collectively attenuating eosinophil production in the bone marrow and recruitment to skin lesions.

### 9.4. Small-Molecule Targeted Drugs

Small-molecule targeted agents, especially JAK inhibitors and tyrosine kinase inhibitors, have emerged as effective oral therapies for moderate-to-severe AD in patients who are refractory to or intolerant of topical treatments and conventional systemic agents. These drugs broadly suppress multiple cytokine signaling pathways implicated in AD pathogenesis.

Hagino et al. [88] found that the total eosinophil count (TEC) or eosinophil/lymphocyte ratio decreased concomitantly with the Eczema Area and Severity Index (EASI) score after treatment with the JAK inhibitor upadacitinib. The correlation between TEC transformation with EASI transformation in upadacitinib treatment suggests that eosinophils may contribute to rash and pruritus in AD and could be a target for upadacitinib treatment [89]. In addition, the JAK1 inhibitor abrocitinib reduces eosinophils through inhibiting the expression of IL-4, IL-13, and IgE [90]. Furthermore, a recent study indicated that abrocitinib decreased Th2-, Th1-, and Treg-related cytokines or chemokines, such as IL-5, IL-6, IL-10, CCL17, CCL18, TNF-α, and IL-2Rα [89]. Recently, ruxolitinib (JAK1/JAK2 inhibitor) has acted as a new FDA-approved drug for moderate-to-severe AD [91]. Furthermore, tyrosine kinase inhibitors, such as imatinib (IMT), are employed clinically to mitigate eosinophil count by targeting specific tyrosine kinases, thereby demonstrating therapeutic efficacy in the management of AD [91]. Seshimo et al. [92] found that IMT suppresses the expression of IL-13, IL-33, and TSLP in the MC903-induced AD-like mice models, thereby inhibiting the activation and recruitment of eosinophils and alleviating AD-like symptoms in mice.

### 9.5. Biologics

Biologics are recombinant proteins, usually monoclonal antibodies, that target specific cytokines or their receptors with high specificity. They are primarily indicated for patients with moderate-to-severe AD whose disease cannot be adequately controlled with topical therapies alone, mainly targeting IL-4/IL-13, IL-5, or IL-33 pathways to regulate eosinophil-driven inflammation. Dupilumab, an IL-4Rα and IL-13R antagonist, reduces eosinophil accumulation by inhibiting IL-4 and IL-13 signaling through binding to the IL-4Rα subunit of the IL-4 and IL-13 co-receptor on the cell surface [93,94,95]. In addition, it can also reduce the level of IgE in AD patients [96]. As fully human IgG4 monoclonal antibodies, tralokinumab [97], and lebrikizumab [98] selectively target and neutralize IL-13, consequently reducing the aggregation of eosinophils in AD and alleviating inflammatory responses. Guttman-Yassky et al. [99] and Scheerens et al. [100] found that patients treated with tralokinumab and lebrikizumab had significantly lower serum IgE levels. Accordingly, in addition to inhibiting the generation and aggregation of eosinophils, these biologics reduce the levels of IgE in patients with AD and alleviate the symptoms of AD as well. Similarly, targeting upstream alarmins has demonstrated significant therapeutic efficacy. For instance, a clinical trial found that treatment with the anti-IL-33 antibody etokimab significantly decreased both peripheral blood eosinophil counts and EASI scores in AD patients [101,102]. Furthermore, Peng et al. [103] found that DNCB-induced AD-like mice treated with an anti-IL-33 antibody exhibited improved clinical symptoms alongside a significant reduction in localized eosinophil infiltration and serum IgE levels. In contrast, biologics directly targeting the IL-5 pathway (e.g., mepolizumab and benralizumab), which represent the most specific approach for eosinophil modulation, have yielded disappointing results [104,105]. Clinical trials have shown that while IL-5 inhibition effectively reduces blood eosinophil counts, it does not consistently lead to significant clinical improvement in AD patients [106]. IL-5 inhibitors are highly effective in asthma but not in AD, suggesting fundamental mechanistic differences between the two eosinophilic diseases, a point we will explore further in the discussion.

### 9.6. Natural Products

Some natural products, especially herbal extracts, have positive effects on eosinophils in AD. Wu et al. [107] found that systemic administration of sclareol reduces the local concentrations of pro-inflammatory cytokines in AD-like lesions, such as IL-6, IL-1β, TNF-α, IL-4, IFN-γ, and IL-17A, thereby inhibiting the recruitment of eosinophils. Topical co-administration of licorice and Portulaca oleracea extracts significantly inhibited skin inflammatory cell infiltration and reduced serum IgE and IL-4 levels in an AD-like murine model [108]. In addition, Hudz et al. [109] found that menthol or menthol derived from peppermint has immunomodulatory effects that can reduce plasma IL-4 as well as eosinophil levels, thereby suppressing itch symptoms. Zhao et al. [110] found that the Fangji Dihuang formulation inhibits DNCB-induced AD-like mice by inhibiting the IL-17 signaling pathway, hence suppressing the engagement and recruitment of eosinophils in AD. A recent case report (n = 1) indicated that Daesiho-tang, a traditional herbal medicine, could improve allergic reactions and reduce palate eosinophil expression through synergistic effects [111] (Table 1). Current evidence supporting these natural products derives predominantly from animal models and isolated case reports, and the lack of standardized active ingredient concentrations limits the strength of available data. Controlled studies with adequate sample sizes are needed to establish their therapeutic validity and safety profiles.

## 10. Discussion

AD is a common chronic, relapsing, inflammatory skin disease characterized by multiple immune responses and eosinophil-mediated tissue damage. This discussion synthesizes current insights into eosinophil biology in AD, evaluates therapeutic strategies targeting eosinophilic pathways, and identifies critical challenges and future directions in translating mechanistic findings into clinical practice.

In AD, eosinophils are implicated in inflammatory responses and tissue damage by releasing various cytokines and granule proteins, including MBP, ECP, EDN, and EPX. These processes are predominantly driven by a Th2-skewed immune response, with cytokines such as IL-4, IL-5, and IL-13 playing crucial roles in the production, recruitment, and activation of eosinophils. Upon binding to IgE-antigen complexes via FcεRI and FcεRII, eosinophils undergo degranulation, thereby amplifying Th2-mediated inflammation. Moreover, these granule proteins exacerbate AD inflammation by directly or indirectly damaging keratinocytes and inducing cytokine release. Although the role of eosinophils in AD has been widely acknowledged, discrepancies remain regarding the functions and mechanisms of different eosinophil subsets. For instance, while some studies suggest that eosinophils may exert immunomodulatory effects, others highlight their destructive role in inflammation. Cytokines such as IL-31 are implicated in the crosstalk between eosinophils and neurons, although their precise mechanisms and contributions to the pathophysiology of AD remain to be elucidated.

Clinically, the counts and percentages of eosinophils positively correlate with the severity of AD in patients. Moreover, eosinophil-related indices and biomarkers serve as prognostic indicators for disease outcomes in patients with AD. Typically, TCS suppresses eosinophils via IL-5/CCL11 inhibition and adhesion blockade. The therapeutic outcomes of biologics reveal a key principle: interventions that target the core of the Th2 inflammatory axis yield more pronounced efficacy. Dupilumab, which targets the shared receptor subunit, simultaneously blocks both IL-4 and IL-13 signaling, thereby broadly suppressing multiple pathogenic processes at their source, including Th2 polarization, IgE class switching, eosinophil recruitment, and epidermal barrier disruption. As a result, it demonstrates the most robust clinical efficacy. In comparison, therapies targeting IL-13 alone show a relatively narrower effect, while the failure of anti-IL-5 agents (e.g., mepolizumab, benralizumab) proves to be the most revealing. However, a notable paradox in AD therapeutics is the discordance between the reduction in blood eosinophil counts and the clinical efficacy of certain biologic agents. For instance, IL-5-targeted biologics such as mepolizumab and benralizumab, which are highly effective in severe eosinophilic asthma and hypereosinophilic syndromes, have failed to demonstrate significant clinical improvement in AD despite markedly depleting circulating eosinophils. Asthma is predominantly driven by systemic, circulating eosinophilia that heavily depends on IL-5 for survival and recruitment. In contrast, AD is a highly heterogeneous disease driven by multiple immune axes beyond Th2, including Th22 and Th17 pathways, which are not directly affected by IL-5 blockade. Therefore, the future of eosinophil-targeting therapies in AD likely lies not in universal depletion, but in combination strategies that simultaneously inhibit upstream drivers of type 2 inflammation (e.g., IL-4/IL-13 blockade with dupilumab) or in identifying specific AD endotypes where eosinophils play a more dominant pathogenic role. Antihistamines mitigate eosinophil activation and migration by inhibiting the expression of GM-CSF, TNF-α, IL-8, and CD11b. Immunosuppressants like cyclosporine A and tacrolimus reduce eosinophil chemotaxis by downregulating CCR3/CCL5. Small-molecule drugs like JAK inhibitors and tyrosine kinase inhibitors broadly inhibit Th2 signaling pathways and IgE expression, normalizing eosinophil counts rapidly. Biologics and natural products achieve significant eosinophil reduction and IgE normalization.

At present, biologics and JAK inhibitors have emerged as the predominant treatment strategies for moderate-to-severe AD. These pharmacological agents are capable of not only effectively attenuating eosinophil levels but also significantly ameliorating patients’ symptoms and enhancing their quality of life. Notably, biologics such as dupilumab, tralokinumab, and lebrikizumab and JAK inhibitors like upadacitinib, ruxolitinib, and abemaciclib have garnered FDA approval for the treatment of AD, while those such as mepolizumab, benralizumab, and etokimab have been evaluated in clinical trials. These agents can not only curtail the production and recruitment of eosinophils but also suppress the expression of IgE, thereby further mitigating AD symptoms. Looking ahead, the therapeutic landscape for AD is poised to prioritize individualized and precision-oriented approaches, such as the implementation of combination therapy regimens in clinical settings and the development of novel, targeted therapeutic agents.

Despite notable progress, several critical gaps remain: Firstly, the long-term safety profiles of biologics and JAK inhibitors require further validation, particularly regarding infection risks and comorbidities. Secondly, natural products demand rigorous pharmacokinetic studies and biomarker-guided optimization. Finally, while current therapies achieve significant eosinophil reduction, their limitations, including heterogeneous responses, safety concerns, incomplete comorbidity control, etc., underscore the need for precision approaches. Future research must unravel eosinophils’ subset diversity, optimize biomarker-guided therapies, and explore regenerative strategies to restore immune balance without compromising host defense.

Emerging technologies are reshaping our understanding of eosinophil heterogeneity and function in AD. Single-cell transcriptomic analyses of lesional skin have revealed previously unrecognized subsets of eosinophils with distinct gene expression profiles, including populations with immunoregulatory or tissue-remodeling phenotypes [115]. Furthermore, the concept of tissue-resident eosinophils, long-lived cells that persist in the skin independently of bone marrow recruitment, challenges the traditional view of eosinophils as short-lived, terminally differentiated effector cells. These resident eosinophils may play a homeostatic role and respond differently to therapeutic interventions. Finally, recent studies suggest that eosinophil function is intimately linked to cellular metabolism [116]; metabolic reprogramming (e.g., shifts in glycolysis or fatty acid oxidation) can dictate eosinophil activation, survival, and effector function [117]. Understanding these nuanced aspects of eosinophil biology may unlock novel therapeutic strategies that selectively modulate pathogenic eosinophil activities while preserving beneficial homeostatic functions.

## Figures and Tables

**Table 1:** Pharmacological therapies for AD affecting eosinophils.

Name	Type	Eosinophil Target	Eosinophil Function	Route	Clinical Stage in AD	Clinical Trial Related to AD and Eosinophils	Reference
Hydrocortisone	Glucocorticosteroids	IL-5 and GM-CSF	Decrease the production and recruitment of eosinophils	Topical	FDA-approved		[71,73,74]
Furoate mometasone		CCL5 and CCL11	Decrease the production and recruitment of eosinophils	Topical	FDA-approved		[72]
Clobetasol propionate		VCAM-1/eotaxin-1	Decrease the production and recruitment of eosinophils	Topical	FDA-approved		[50]
Loratidine	Antihistamines	H_1_R, GM-CSF, TNF-α, and IL-18	Decrease the production, recruitment and degranulation of eosinophils	Oral	/	/	[78,79]
Izuforant		H_4_R, CD11b, ICAM-1, and IL-18	Decrease the recruitment and degranulation of eosinophils	Oral	Clinical Stage in AD	NCT04853992 (phase 2, completed)	[77,82]
Cyclosporin A	Immunosuppressants	CD11b	Decrease the production and recruitment of eosinophils	Oral	Clinical Stage in AD	NCT00809172 (phase 3, terminated)	[85]
Tacrolimus		Calcineurin	Decrease the recruitment of eosinophils	Topical	FDA-approved		[70]
Upadacitinib	Small-molecule targeted drugs	JAK1	Decrease the recruitment of eosinophils	Oral	FDA-approved		[88,89]
Ruxolitinib		JAK1/JAK2	Decrease the recruitment of eosinophils	Oral	FDA-approved		[102]
Abrocitinib		JAK1	Decrease the recruitment of eosinophils	Oral	FDA-approved		[91,112]
Imatinib		Protein tyrosine kinase (PTK)	Decrease the recruitment of eosinophils	Oral	/	/	[92]
Dupilumab	Biologics	IL-4 and IL-13	Decrease the recruitment of eosinophils	Subcutaneous injection	FDA-approved		[93,94,95,113]
Tralokinumab		IL-13	Decrease the recruitment of eosinophils	Subcutaneous injection	FDA-approved		[97,99]
Lebrikizumab		IL-13	Decrease the recruitment of eosinophils	Subcutaneous injection	FDA-approved		[98,100]
Mepolizumab		IL-5	Decrease the production and recruitment of eosinophils	Subcutaneous injection	Clinical Stage in AD	NCT03055195 (phase 2, terminated)	[106]
Benralizumab		IL-5	Decrease the production and recruitment of eosinophils	Subcutaneous injection	Clinical Stage in AD	NCT04605094 (phase 2, terminated)	[114]
Etokimab		IL-33	Decrease the recruitment of eosinophils	Subcutaneous injection	Clinical Stage in AD	NCT03533751 (phase 2, terminated)	[101]
Glycyrrhizin	Natural products	Th2-related cytokines	Decrease the recruitment of eosinophils	Topical	/	/	[108]
Menthol		Th2-related cytokines	Decrease the recruitment of eosinophils	Topical	/	/	[109]
Fangji Dihuang formulation		Th17-related cytokines	Decrease the recruitment of eosinophils	Oral	/	/	[110]
Daesiho-tang		Th1/Th2-related cytokines	Decrease the production and recruitment of eosinophils	Oral	/	/	[111]

FDA: Food and Drug Administration; IL: interleukin; JAK: Janus kinase; AD: atopic dermatitis; PTK: protein tyrosine kinase.

## Data Availability

No datasets were generated or analyzed during the current study.

## Abbreviations

AD	atopic dermatitis
AA	asthma
AR	allergic rhinitis
EoPs	eosinophil precursors
GM-CSF	granulocyte–macrophage colony-stimulating factor
IL-5R	interleukin-5 receptor
CCL	chemo-attractant cytokine ligand
TSLP	thymic stromal lymphopoietin
BNDF	brain-derived neurotrophic factor
IKKβ	nuclear factor-κB kinase subunit β
H4R	histamine H4 receptor
MBP	major basic protein
ECP	eosinophilic cationic protein
EDN	eosinophilic-derived neurotoxin
EPX	eosinophilic peroxidase
VEGF	vascular endothelial growth factor
Bregs	regulatory B cells
Tregs	regulatory T cells
NGF	nerve growth factor
PAR2	protease-activated receptor 2
ILC2s	group 2 innate lymphoid cells
CTGF	connective tissue growth factor
CCR3	CC chemokine receptor 3
PSGL-1	P-selectin glycoprotein ligand 1
PF4	platelet factor 4
TCSs	topical glucocorticoids
IFN-α	interferon-alpha
TEC	total eosinophils count
H2R	histamine 2 receptor
H1R	histamine 1 receptor
IMT	imatinib
EASI	Eczema Area and Severity Index
JAK	janus kinase
DCs	dentric cells
PTK	protein tyrosine kinase
